# Editorial: HIV Latency: novel insights into the viral reservoir and therapeutic strategies

**DOI:** 10.3389/fcimb.2024.1434507

**Published:** 2024-06-14

**Authors:** Ana Carolina Soares de Oliveira, Celina Monteiro Abreu

**Affiliations:** ^1^ Division of Clinical Immunology and Allergy, School of Medicine, University of São Paulo, São Paulo, Brazil; ^2^ Laboratory of Molecular Evolution and Bioinformatics, Department of Microbiology, Biomedical Sciences Institute, University of São Paulo, São Paulo, Brazil; ^3^ Department of Molecular and Comparative Pathobiology, Johns Hopkins University, Baltimore, MD, United States

**Keywords:** HIV latency, cellular metabolism, biomarkers, preventive strategies, HIV reservoir

HIV cure and prevention are two critical aspects of combating the HIV/AIDS epidemic. Antiretroviral therapy (ART) has transformed HIV from a deadly to a chronic disease. However, HIV can remain dormant in specific cells and become active again if the therapy is stopped. This latent virus is mainly found in CD4+ T cells and macrophages transitioning from active to resting. The virus can establish itself in these cells very early during acute infection, and even under suppressive ART, the HIV reservoir remains dynamic, with low-level replication and seeding through clonal expansion (Lau et al., 2021). To understand the cellular reservoir in individuals living with HIV who have an undetectable viral load, it is crucial to identify specific latency biomarkers.

Zhang et al. employed a single-cell methodology to pinpoint markers of HIV latency, enabling direct comparison between gene expression profiles of HIV RNA-positive and negative cells within the same sample. Notably, eleven out of the seventeen identified genes (LMNA, IL2RA, ZMAT3, RGS16, PMEPA1, IER5, GATA3, PHPT1, PLEC, AMNAIL, and NEAT1) were validated as biomarkers of resting cells latently infected with HIV in the models used. It is important to recognize that certain gene expression profiles may overlap between productive and latent infections, necessitating careful selection of shared genes when developing strategies for latency eradication. The study does have limitations, particularly in identifying cells harboring transcriptionally silent, integrated HIV DNA. The authors have proposed several candidates as biomarkers of HIV latency; nonetheless, further research is needed to explore these biomarker candidates across diverse HIV populations to validate their full utility.

Other latency studies are essential to the development of HIV cure strategies and focus on latency mechanisms. The size of the reservoir is determined by various factors, such as the host’s immune response, cellular metabolism, and ART initiation. Two review papers explored the HIV infection and latency mechanisms: noncanonical NF-kB and cellular metabolism. Chandrasekar et al. describe the importance of the latency mechanism via the NF-Kb pathway and its implications for the development of new strategies for “shock and kill” HIV cure. The canonical path triggers immune cell activation, proinflammatory cytokines, and angiogenesis, leading to immune recruitment. The noncanonical NF-κB pathway involves the NF-κB2 protein in a p52: RelB heterodimer that establishes HIV latency. This pathway may be more favorable for HIV latency because it is a slow and persistent process that does not lead to global T-cell activation, allowing the virus to remain hidden from the immune system. Additionally, it has been demonstrated that cells with lower levels of NF-κB, mostly mediated through the noncanonical p50 pathway, may facilitate the establishment of HIV latency.​​ The authors conclude that further studies are needed to identify specific agonists of this pathway, which significantly enhance the “shock and kill” approach, contributing to advances in the HIV cure field.

Another important aspect of HIV latency is understanding cellular metabolism; HIV has a profound impact on the cellular metabolism within CD4+ T cells, resulting in altered metabolic pathways and mitochondrial dysfunction (Hollenbaugh et al., 2011). The virus uses processes like glycolysis and glutaminolysis to fuel its replication cycle (Akiso et al., 2023). Furthermore, dysregulated lipid metabolism exacerbates immune dysfunction and inflammation observed in HIV/AIDS. It is vital to understand these metabolic intricacies to identify potential drug targets and craft innovative therapeutic interventions against HIV/AIDS. In their review, Crater et al. described the importance of cellular metabolism and latency strategies for the cure. HIV-1 is intricately connected with host cell metabolism throughout its replicative cycle, with mTOR as a crucial regulator. mTOR and its downstream effectors modulate glycolysis, lipid metabolism, and other pathways essential for viral replication and latency. Pre-clinical studies using rapamycin or analogs in animal models have shown promise, with ATP-competitive mTOR kinase inhibitors exhibiting protective effects against HIV infection in humanized mice. However, studies on Akt/mTOR activation as a latency-reversing agent (LRA) have yielded limited success *in vivo*. Other studies exploring indirect mTOR inhibitors, like metformin, have shown nuanced outcomes, with no changes in transcriptionally competent reservoirs observed in peripheral blood CD4+ T cells. While analogs and mTOR inhibitors have yet to demonstrate efficacy in reducing viral reservoirs *in vivo*, the significance of cell metabolism in HIV replication and latency underscores its relevance in new crafting HIV-1 cure strategies, but future clinical investigations must consider age and sex, given their significant roles in regulating inflammation and metabolism.

The advancement of preventive strategies is a pivotal aspect of the global endeavor to mitigate HIV infection rates. Controlling the spread of HIV in the non-infected population, particularly among young women who are increasingly affected, remains a significant challenge in HIV research. Vaginal or rectal administration of pharmaceuticals and PrEP products has several advantages. Local absorption of drugs results in enhanced bioavailability and high concentrations at the site of viral entry while limiting systemic exposure, decreasing systemic side effects, and increasing adherence (Peet et al., 2019). Thurman et al. described the need for multi-purpose prevention technology (MPT) products that could prevent both human immunodeficiency virus (HIV) and herpes simplex virus type 2 (HSV2). The Phase I study evaluated a fast-dissolve insert that could be used vaginally or rectally to prevent infection. Sixteen women applied one TAF (20mg)/EVG (16mg) vaginal insert and were split into two groups for sample collection. The study also measured the concentration of EVG, TAF, and tenofovir (TFV) in plasma, vaginal fluid, and tissue, as well as the concentration of TFV-diphosphate (TFV-DP) in vaginal tissue. The TAF/EVG insert was safe, with all treatment-emergent adverse events graded as mild and acceptable to participants. The development of microbicides and pre-exposure prophylaxis (PrEP) products that are safe, effective, and acceptable can help reduce HIV sexual transmission in young women.

Efforts to achieve an HIV cure and prevent new infections are multifaceted and require a combination of biomedical, behavioral, and structural interventions. While challenges remain, ongoing research and global initiatives aimed at expanding access to prevention and treatment services offer hope for eventually ending the HIV/AIDS epidemic. Continued investment in research, innovation, and community engagement is essential to realizing the goal of an AIDS-free generation.

## Author contributions

AO: Writing – original draft, Writing – review & editing. CA: Writing – original draft, Writing – review & editing.
